# Effect of Oat β-Glucan Intake on Glycaemic Control and Insulin Sensitivity of Diabetic Patients: A Meta-Analysis of Randomized Controlled Trials

**DOI:** 10.3390/nu8010039

**Published:** 2016-01-13

**Authors:** Xiao Li Shen, Tao Zhao, Yuanzhong Zhou, Xiuquan Shi, Yan Zou, Guohua Zhao

**Affiliations:** 1 School of Public Health, Zunyi Medical University, Zunyi 563000, Guizhou, China; xiaolishen1983@163.com (X.L.S.); 15186613384@163.com (T.Z.); zhouyuanzhong@163.com (Y.Z.); xqshi@zmc.edu.cn (X.S.); zouyan54321@126.com (Y.Z.); 2 College of Food Science, Southwest University, Chongqing 400716, China; 3 Chongqing Engineering Research Centre of Regional Foods, Chongqing 400716, China

**Keywords:** oat β-glucan, diabetes mellitus, glycaemic control, insulin sensitivity, meta-analysis

## Abstract

Many individual studies on oat β-glucan (OBG) confirmed its functionality in improving type 2 diabetes mellitus (T2DM), but disagreements were identified among those results. To derive a pooled estimate of these results, relevant articles, published before 5 September 2015, were collected from four electronic databases (Pubmed, Cochrane Library, Scopus, and Web of Science) and subjected to meta-analysis in the present work. In total, four articles, dealing with 350 T2DM patients combined, met the inclusion criteria. Compared to control, T2DM patients administrated OBG from 2.5 to 3.5 g/day for 3 to 8 weeks presented significantly lowered concentrations in fasting plasma glucose (FPG) by −0.52 (95% CI: −0.94, −0.10) mmol/L (*p* = 0.01) and glycosylated hemoglobin (HbA_1c_) by −0.21% (95% CI: −0.40, −0.02) (*p* = 0.03). However, OBG intake did not significantly lower the fasting plasma insulin (FPI) concentration. In conclusion, mediate-term OBG intake (3–8 weeks) favored the glycaemic control of T2DM patients but did not improve their insulin sensitivity. Regrettably, data upon the effects of long-term OBG intake on glycaemic control and insulin sensitivity were scarce, which is of much importance and should be addressed in future research.

## 1. Introduction

Diabetes mellitus (DM) is a group of metabolic diseases characterized by hyperglycemia resulting from defects in insulin secretion, insulin action, or both [1]. It had been reported that 382 million people had diabetes in 2013. Moreover, this number is expected to increase to 592 million by 2035 in the worldwide. However, the greatest increase in diabetes prevalence is expected to occur in low- and middle-income countries over the next 22 years. China has the highest number of adults with diabetes (over 98.4 million, account for about 25.8% of the total number of diabetes in the worldwide) in 2013, and this number is expected to increase to 142.7 million (account for about 24.1% of the total number of diabetes in the worldwide) by 2035 [2]. However, there is no fine cure for DM [3], and the frequent use of medications is expensive and the side effects are difficult to avoid [4]. Lifestyle modification is the first-line management for Type 2 diabetes mellitus (T2DM) [5]. A healthy diet, a normal body weight maintenance, and regular physical activity can prevent or mitigate the harmful effects of T2DM [4,6].

In the mid-1980s, oat was recognized as a healthy food, contributing to the prevention of heart disease, after which it became more popular in human nutrition. The common oat (*Avena sativa*) is unique among the cereals not only due to its nutritional profile, but also due to its medicinal value against various maladies. To some extent, these outstanding functional properties of oat can not deviate from β-glucan [7]. Oat β-glucan (OBG), the important soluble dietary fiber found in oats, is mainly located in the endosperm cell walls (75%) and bran (10.4%) [7]. Purified OBG is linear non-starchy polysaccharide composed of d-glucopyranosyl units linked by a mixture of 70% β-(1 → 4) and 30% β-(1 → 3) glycosidic linkages [6,7]. Furthermore, OBG is also unique. For example, OBG has a higher molecular weight than barley β-glucan [6] and has high viscosity even at relatively low concentrations (1%) [7]. The attenuation of postprandial plasma glucose and insulin responses in people with or without T2DM is related to the high viscosity of OBG, because increased viscosity delays gastric emptying, slows intestinal transit, and postpones glucose and sterol absorption in the intestine [7]. Therefore, OBG is regarded as a potentially health-promoting food ingredient, and is used as a thickening agent, a stabilizer, and a fat replacer in the food industry [6].

Although health claims associated with oats had been approved by the US Food and Drug Administration (FDA) in 1997, the health-promoting effect of oat products is achieved only when oat products are consumed regularly for some time and reach a certain OBG dose threshold in the diet [6,7]. In 1997, a daily intake of ≥3 g OBG was recommended by the US FDA to obtain a cholesterol-lowering effect [8]. It has also been reported that the consumption of 3 g OBG-enriched bread for 3 weeks can reduce insulin resistance in patients with T2DM [9]. However, another study has found that there are no diet-related effects on fasting plasma glucose, fasting plasma insulin or insulin resistance in patients with T2DM when 3.9 g OBG-enriched meals are consumed for consecutive eight-week periods [5]. Although there is a systematic review that pays attention to the effect of β-glucans in the control of blood glucose levels of diabetic patients [4], it is lacking quantitative assessment. Furthermore, the document retrieval of that systematic review was conducted no later than November 2013 [4]. Therefore, it is necessary to update a meta-analysis of high-quality randomized controlled trials (RCTs) to quantitatively assess whether OBG intake has a beneficial effect on DM and to make some suggestions regarding diabetes diet based on our analysis.

## 2. Methods

### 2.1. Search Strategy

Relevant articles, published before 5 September 2015, were searched from four electronic databases (Pubmed, Cochrane Library, Scopus, and Web of Science). The search strategy was implemented using the following keywords: oat and diabetes. Review articles were filtered out, and publishing languages were limited to English and Chinese (at least with English abstracts in the above databases). With one exception, it could not restrict publishing languages to English and Chinese in the database of the Cochrane Library.

### 2.2. Inclusion and Exclusion Criteria

The inclusion criteria (IC) of the meta-analysis were as follows [3,8]: (1) randomized controlled trials (RCTs) conducted in diabetic patients with either a cross-over or parallel design; (2) use of any type of oat-enriched diet in the intervention group; (3) use of oat as the only acceptable source of β-glucan; (4) inclusion of an appropriate control group; and (5) use of data with available means and any of standard deviations, standard errors, or 95% CI as the endpoint values for fasting plasma glucose concentration, fasting plasma insulin concentration, or HbA_1c_(%).

The exclusion criteria (EC) of the meta-analysis were as follows [3,8]: (1) that the amount of OBG is not declared/measured; (2) that the effect of OBG cannot be isolated from additional ingredient in combination diet; (3) a treatment period less than 2 weeks; (4) an inappropriate control group, e.g., containing a small quantity of OBG; (5) outcome measurement not containing one of the following: fasting plasma glucose concentration, fasting plasma insulin concentration, and HbA_1c_(%); (6) animal studies; (7) secondary information, e.g., review articles, editorials, commentaries; and (8) publishing languages not English or Chinese.

### 2.3. Study Selection

An initial screening for potentially relevant studies was undertaken independently by two authors (X. L. Shen and T. Zhao). All retrieved studies were evaluated according to the inclusion and exclusion criteria. All the disagreements were resolved by consensus after discussion with a third researcher (G. Zhao).

### 2.4. Methodological Assessment

The risk of bias was assessed according to the Cochrane risk-of-bias tool. The assessment mainly contains six domains of bias: selection bias, performance bias, detection bias, attrition bias, reporting bias, and other bias [10]. Studies were regarded as having a high risk of bias if the methodological flaw was likely to have affected the true outcome, a low risk if the study’s methodological flaw was considered as unimportant to the true outcome, and an unclear risk if insufficient information was provided to judge risk of bias [11]. Critical appraisal was carried out independently by two authors (X. L. Shen and T. Zhao). All the differences were resolved by consensus via discussion with a third researcher (Y. Zhou).

### 2.5. Data Extraction

Detailed information from all included studies for the meta-analysis was extracted independently by two authors (X. L. Shen and T. Zhao). All the discrepancies were resolved through a discussion with a third author (Y. Zhou). Effective data of fasting plasma glucose concentration, fasting plasma insulin concentration, and HbA_1c_(%) were extracted from all included trials for the meta-analysis. The standard deviations for changes from baseline were obtained from Chapters 7.7.3.2 and 16.1.3.2 of the Cochrane Handbook, version 5.1.0 [12] if they could not be extracted from the study directly. The detailed information collected from each included study was as follows: first author; publication year; sample size; healthy status, gender ratio, age, and BMI of participants; molecular weight of OBG; trial design (parallel or crossover); details of OBG treatment group and control group; run-in period and/or washout period of crossover design; and intervention period.

### 2.6. Statistical Analysis

Review manager 5.3 was used for the statistical analysis. The statistical heterogeneity was assessed by the chi-squared test (*p* < 0.1), and *I*^2^ was included in the forest plots [12]. The random-effects model was used if the overall pooled studies showed significant heterogeneity. Otherwise, a fixed-effects model was used. Funnel plot was used to observe publication bias [3].

## 3. Results

### 3.1. Literature Search

**Figure 1:**
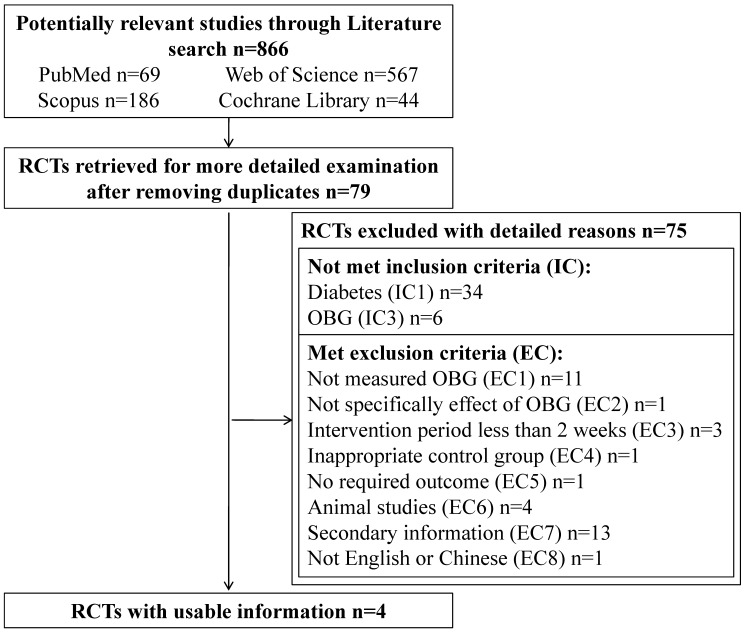
Flow diagram for the selection of randomized controlled trials (RCTs) for the meta-analysis. IC1: RCTs conducted in diabetic patients with either a cross-over or parallel design. IC3: use of oat as the only acceptable source of β-glucan. EC1: the amount of oat β-glucan (OBG) is not declared/measured. EC2: the effect of OBG cannot be isolated from additional ingredient in combination diet. EC3: treatment period less than 2 weeks. EC4: inappropriate control group, e.g., contain a small quantity of OBG. EC5: outcome measurement not containing one of the following: fasting plasma glucose concentration, fasting plasma insulin concentration, and HbA_1c_(%). EC6: animal studies. EC7: secondary information, e.g., review article, editorial, commentary. EC8: publishing languages is not English or Chinese.

Flow diagram for the selection of RCTs for the present meta-analysis is shown in Figure 1. The initial literature search from four databases (Pubmed, Cochrane Library, Scopus, and Web of Science) yielded 866 publications before removing duplicates. Based on titles and abstracts, 79 articles were retrieved to satisfy the standard of RCTs after removing duplicates. Of these, 75 articles were excluded as they did not meet the inclusion criteria (IC) or the exclusion criteria (EC). According to IC1, IC3, and EC1-8, the number of excluding publications is 34, 6, 11, 1, 3, 1, 1, 4, 13, and 1, respectively. More detailed information about the 75 articles is provided in Supplementary A. At last, four articles were eligible for the present meta-analysis [9,13,14,15].

### 3.2. Study Characteristics

The characteristics of the four RCTs included in the present meta-analysis are described in Table 1. The total number of patients with T2DM in four studies was 350. One RCT had a cross-over design and three had a parallel design. The dose of OBG ranged from 2.5 to 5.0 g/day, and the intervention period ranged from 3 to 8 weeks. Two doses of OBG (2.5 and 5.0 g/day) were used in the study of Ma *et al.* [15], in order to increase the comparability with the other three studies, only the dose of 2.5 g/day OBG was selected to include into the present meta-analysis. Thus, the dose of OBG was narrowed to range from 2.5 to 3.5 g/day for the present meta-analysis.

A risk of bias graph and summary according to the review authors’ judgements about each risk of bias item for each included study are presented in Figure S1a,b.

**Table 1:** Characteristics of the four included randomized controlled trials.

Author (Publication Year)	No. of Subjects	Healthy Status	Male (%)	Age (Years) Mean (or Range)	BMI (kg/m^2^) Mean (or Range)	Molecular Weight of OBG (kDa)	Trial Design	Treatment Group	Control Group	Run-in/Washout Period	Intervention Period
Cugnet-Anceau, C., *et al.* (2010) [13]	53	T2DM	60	61.9 (44–75)	29.82	80	Parallel	Soluble oat soups (3.5 g/day β-glucan)	Soups without β-glucan	3 weeks	8 weeks
Kabir, M., *et al.* (2002) [14]	13	T2DM	100	59 (41–67)	28 (23–36)	Not reported	Cross-over	Oat bran concentrate (3 g/day β-glucan)	Whole wheat grains without β-glucan	1 month/15 days	Two periods of 4 weeks
Liatis, S., *et al.* (2009) [9]	41	T2DM	56	63	28.47	Not reported	Parallel	Bread (3 g/day β-glucan)	White bread without β-glucan	3 weeks	3 weeks
Ma, X., *et al.* (2013) [15]	243	T2DM	43	59.4 (50–65)	26.69	Not reported	Parallel	Organic naked oat with whole germ (2.5 or 5 g/day β-glucan)	Diet without β-glucan	1 week	30 days

Abbreviations: T2DM = Type 2 diabetes mellitus; OBG = oat β-glucan.

### 3.3. Effect of OBG Intake on Fasting Plasma Glucose (FPG) Concentration

**Figure 2:**
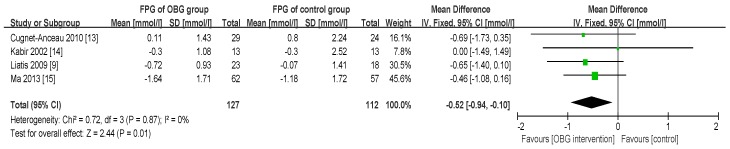
Forest plot for the effect of OBG intake on fasting plasma glucose (FPG) concentration. Data are expressed as mean differences in FPG concentration with 95% confidence intervals (CI). For Ma 2013 [15], FPG concentration is obtained at a dose of 2.5 g/day OBG.

**Figure 3:**
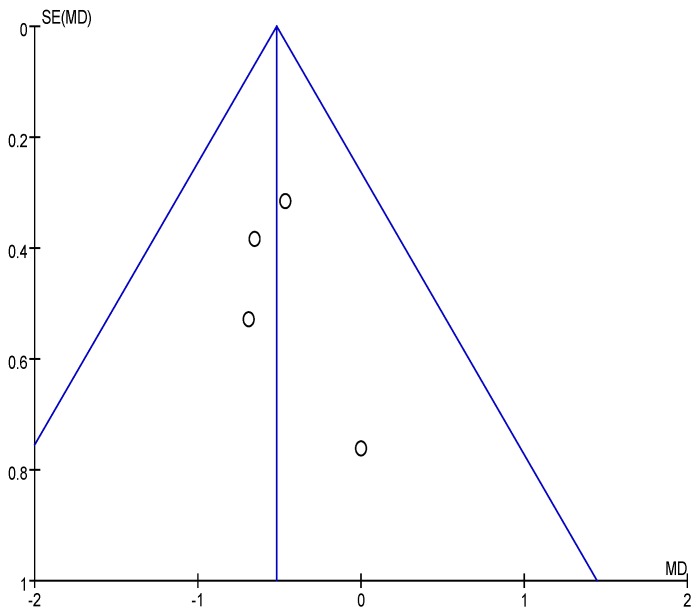
Funnel plot for the effect of OBG intake on fasting plasma glucose (FPG) concentration. For Ma 2013 [15], FPG concentration is obtained at a dose of 2.5 g/day OBG.

The effect of OBG intake on FPG concentration is revealed in the forest plot (Figure 2). No significant heterogeneity was observed among the four studies (χ^2^ = 0.72, *p* = 0.87, *I*^2^ = 0%). The fixed-effects meta-analysis of FPG concentration revealed a statistically significant difference in OBG intake relative to control intake of −0.52 mmol/L (95% CI: −0.94, −0.10; *p* = 0.01). Although the amount of included studies was relatively few, no serious publication bias was observed by the funnel plot (Figure 3).

### 3.4. Effect of OBG Intake on Glycosylated Hemoglobin (HbA_1c_) Concentration

**Figure 4:**
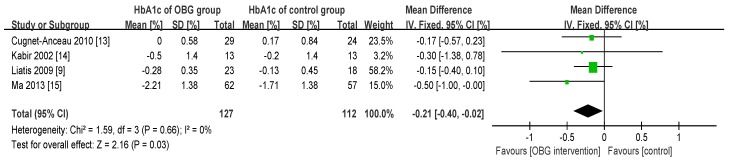
Forest plot for the effect of OBG intake on HbA_1c_(%). Data are expressed as mean differences in HbA_1c_(%) with 95% confidence intervals (CI). For Ma 2013 [15], FPG concentration is obtained at a dose of 2.5 g/day OBG.

**Figure 5:**
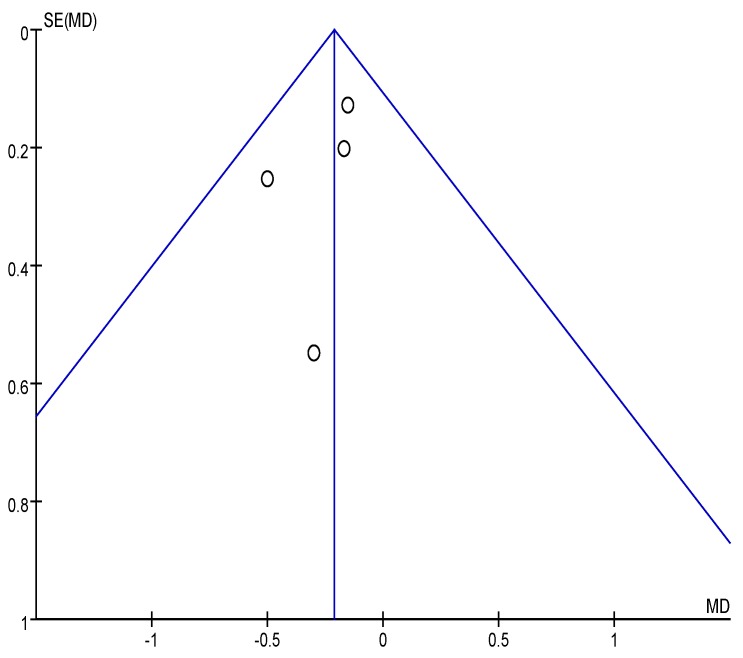
Funnel plot for the effect of OBG intake on HbA_1c_(%). For Ma 2013 [15], HbA_1c_(%) is obtained at a dose of 2.5 g/day OBG.

The effect of OBG intake on HbA_1c_(%) is shown in the forest plot (Figure 4). No significant heterogeneity was revealed among the four studies (χ^2^ = 1.59, *p* = 0.66, *I*^2^ = 0%). The fixed-effects meta-analysis of HbA_1c_(%) displayed a statistically significant difference in OBG intake relative to control intake of −0.21% (95% CI: −0.40, −0.02; *p* = 0.03). Although the amount of included studies was relatively few, no serious publication bias was observed by the funnel plot (Figure 5).

### 3.5. Effect of OBG Intake on Fasting Plasma Insulin (FPI) Concentration

**Figure 6:**
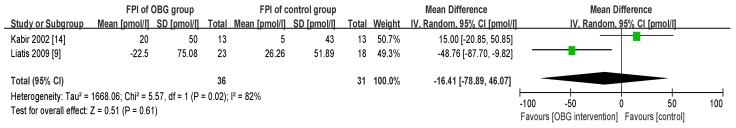
Forest plot for the effect of OBG intake on fasting plasma insulin (FPI) concentration. Data are expressed as mean differences in FPI concentration with 95% confidence intervals (CI). For Ma 2013 [15], FPI concentration is obtained at a dose of 2.5 g/day OBG.

The effect of OBG intake on fasting plasma insulin (FPI) concentration is displayed in the forest plot (Figure 6). Significant heterogeneity was observed between two studies (χ^2^ = 5.57, *p* = 0.02, *I*^2^ = 82%). The random-effects meta-analysis of FPI concentration suggested no significant difference between OBG intake and control intake (*p* = 0.61). As the relevant research was scarce, it is impossible to conduct subgroup analysis.

## 4. Discussion

In the present meta-analysis, OBG intake from 2.5 to 3.5 g/day for 3 to 8 weeks in T2DM patients could obviously improve glycaemic control, such as significantly lowered FPG and glycosylated hemoglobin (HbA_1c_) concentration. No significant heterogeneity nor any serious publication bias was observed with regard to FPG and HbA_1c_ concentration among the four studies. According to the qualitatively systematic review of Andrade *et al.* [4], the intake of β-glucans could decrease the glucose levels of diabetic patients, which agrees with our result of FPG. According to the double-blind RCTs of Kobayakawa *et al.* [16] in Japanese men with mild hyperglycemia and visceral fat obesity, even a moderate amount of dietary fiber (7.5 g/day) intake for 12 weeks may be beneficial for managing the FPG level accompanying insulin resistance, body weight, and body mass index. OBG is reported as a unique water-soluble dietary fiber with high viscosity. Molecular weight, concentration, and distribution of OBG have a great effect on the viscosity. For instance, OBGs extracted from bran have higher viscosities than those from endosperm. In addition, OBGs from different oat varieties have different molecular weights [7]. Furthermore, the viscosity of OBG has an important effect on the health-promoting properties of OBG. Wood *et al.* [17,18] showed that the reductions in glucose and insulin responses after a meal are significantly linear relative to the viscosity of OBG. For another example, the meta-analysis of Whitehead *et al.* [8] led to the conclusion that the addition of OBG with a molecular weight (MW) ≥100 kDa and a dose ≥3 g/day to the diet for 2–12 weeks could reduce LDL and total cholesterol in free-living normocholesterolemic or hypercholesterolemic adults. The suggested mechanism of cholesterol-lowering is related to the viscosity of OBG, which promotes the excretion of bile acids [19]. The OBG with MW <100 kDa displays Newtonian behavior and has a low viscosity at the relevant concentrations [8]. However, only Cugnet-Anceau *et al.* [13] have reported the molecular weight of OBG (80 kDa) among the four included studies of the present meta-analysis. For the study of Cugnet-Anceau *et al.* [13], the concentration of FPG and HbA_1c_ was not reduced with OBG intake, whether if it was related to the relative low molecular weight of OBG (80 kDa). Therefore, it is essential to conduct a large number of finer and higher quality RCTs.

A reduction of 0.52 mmol/L of FPG and 0.21% of HbA_1c_(%) after 3 to 8 weeks of intervention, although rather small in size, could be relevant in well-controlled type 2 diabetic patients. However, the clinical significance of these results remains to be confirmed in the long term, when reduced patient compliance or physiological adaptive changes induced by chronic oat addition may occur. Andrade *et al.* [4] pointed out that the consumption of greater doses of β-glucans or smaller doses of β-glucans for longer periods of time may produce better results.

On the aspect of insulin sensitivity, OBG intake from 2.5 to 3.5 g/day for 3 to 8 weeks in T2DM patients had no significant effect on FPI concentration. At the same time, significant heterogeneity was observed between two studies about FPI. According to the meta-analysis of Bao *et al.* [3], significant reductions of FPI concentrations in healthy individuals or metabolic disease patients were observed only in the subgroups (5 participants) of long-term studies (≥8 weeks), but not in those (5 participants) of short-term studies (<8 weeks). On the whole, the high-quality studies in regard to the effect of oat intake on FPI concentration were scarce, whether in healthy individuals or in diseased patients.

However, the recent meta-analysis of Bao *et al.* [3] concluded that FPI was significantly lowered with oat intake, but FPG and HbA_1c_(%) were not significantly decreased. This conclusion was the opposite of our meta-analysis. The possible reasons are mainly as follows: The subjects contained healthy people, T2MD patients, overweight individuals, and so on in the meta-analysis of Bao *et al.*, but only T2MD patients were concerned specifically in our meta-analysis; moreover, the intervention period varied from 2 h to 16 weeks in the meta-analysis of Bao *et al.*, but the span of intervention period (3 to 8 weeks) was smaller in our meta-analysis.

The present meta-analysis has some limitations. The sample sizes of relative long-term and high-quality RCTs researching the effect of OBG intake on glycaemic control and insulin sensitivity are extremely small. Furthermore, among the available studies, the reported outcomes referring to the effect of OBG intake on FPI concentration is rather limited, which could have severely restricted outcome assessment. Lastly, language bias is unavoidable, since articles that were not published in English or Chinese were excluded.

## 5. Conclusions

OBG intake from 2.5 to 3.5 g/day for 3 to 8 weeks in T2DM patients could obviously improve glycaemic control, such as significantly lowered fasting plasma glucose (FPG) and glycosylated hemoglobin (HbA_1c_) concentration. On the aspect of insulin sensitivity, OBG intake from 2.5 to 3.5 g/day for 3 to 8 weeks in T2DM patients had no significant effect on fasting plasma insulin (FPI) concentration.

## Supplementary Files

Supplementary File 1
