# Supplementary material for: Effect of Oat β-Glucan Intake on Glycaemic Control and Insulin Sensitivity of Diabetic Patients: A Meta-Analysis of Randomized Controlled Trials
Source: Nutrients. 2016 Jan 13;8(1):39. doi: 10.3390/nu8010039 (PMC4728652; doi:10.3390/nu8010039)
Supplement: Supplementary File 1 [file nutrients-08-00039-s001.docx]

Supplementary Materials: Effect of Oat β-Glucan Intake on Glycaemic Control and Insulin Sensitivity of Diabetic Patients: A Meta-Analysis of Randomized Controlled Trials

Xiao Li Shen, Tao Zhao, Yuanzhong Zhou, Xiuquan Shi, Yan Zou and Guohua Zhao

|  |
| --- |
| (**a**) |
|  |
| (**b**) |

**Figure S1.** (**a**) Risk of bias graph according to review authors’ judgements about each risk of bias item presented as percentages across all included studies; (**b**) Risk of bias summary according to review authors’ judgements about each risk of bias item for each included study.

Supplementary A. RCTs Excluded with Detailed Reasons

Not met inclusion criteria (IC):

Diabetes (IC1) [[1–34](#_ENREF_1)]

OBG (IC3) [[35–4](#_ENREF_35)0]

Met exclusion criteria (EC):

Not declared/measured OBG (EC1) [[41–5](#_ENREF_42)1]

Not specifically effect of OBG (EC2) [[5](#_ENREF_53)2]

Intervention period less than 2 weeks (EC3) [[53–5](#_ENREF_54)5]

Inappropriate control group (EC4) [[5](#_ENREF_57)6]

No required outcome (EC5) [[5](#_ENREF_58)7]

Animal studies (EC6) [[58–6](#_ENREF_59)1]

Secondary information (EC7) [[62–7](#_ENREF_63)4]

Not English or Chinese (EC8) [[7](#_ENREF_76)5]

References

1. Kruk, M.; Kadziela, J.; Reynolds, H.R.; Forman, S.A.; Sadowski, Z.; Barton, B.A.; Mark, D.B.; Maggioni, A.P.; Leor, J.; Webb, J.G.; *et al*. Predictors of outcome and the lack of effect of percutaneous coronary intervention across the risk strata in patients with persistent total occlusion after myocardial infarction: Results from the OAT (Occluded Artery Trial) study. *JACC. Cardiovasc. Interv.* **2008**, *1*, 511–520.
2. Liljeberg, H.G.; Granfeldt, Y.E.; Björck, I.M. Products based on a high fiber barley genotype, but not on common barley or oats, lower postprandial glucose and insulin responses in healthy humans. *J. Nutr.* **1996**, *126*, 458–466.
3. Reynolds, H.R.; Forman, S.A.; Tamis-Holland, J.E.; Steg, P.G.; Mark, D.B.; Pearte, C.A.; Carvalho, A.C.; Sopko, G.; Liu, L.; Lamas, G.A.; *et al*. Relationship of female sex to outcomes after myocardial infarction with persistent total occlusion of the infarct artery: Analysis of the Occluded Artery Trial (OAT). *Am. Heart J.* **2012**, *163*, 462–469.
4. Stentz, F.B.; Hutson, M.L.; Tylavsky, F.A.; Kitabchi, A.E. Development of a randomized trial using a high carbohydrate (HC) or high protein (HP) diet intervention that furnishes ready-to-eat foods and beverages available online or in local grocery stores. *Diabetes* **2010**, *59*, A679–A679.
5. White, H.D.; Reynolds, H.R.; Carvalho, A.C.; Liu, L.; Pearte, C.A.; Dzavik, V.; Kruk, M.; Steg, P.G.; Lamas, G.A.; Hochman, J.S. Predictors of reinfarction following PCI or medical management using the universal definition in patients with total occlusion after myocardial infarction: Results from OAT long term follow up. *Eur. Heart J.* **2011**, *32*, 738–739.
6. Bosch, M.-A.; Hernandez, J.-A.; Serra-Prat, M.; Rio, N.D.; Santamaria, A.; Remacha, A.; Clapes, V.; Lopez, R.; Oliveira, A.-C.; Mateo, J.; *et al*. Effectiveness and Safety of Oral Anticoagulant Therapy in Patients with Non-Rheumatic Atrial Fibrillation. *Blood* **2002**, *100*, 1067.
7. Hallikainen, M.; Toppinen, L.; Mykkanen, H.; Agren, J.J.; Laaksonen, D.E.; Miettinen, T.A.; Niskanen, L.; Poutanen, K.S.; Gylling, H. Interaction between cholesterol and glucose metabolism during dietary carbohydrate modification in subjects with the metabolic syndromes. *Am. J. Clin. Nutr.* **2006**, *84*, 1385–1392.
8. Hochman, J.S.; Grp, O.A.T.R. The occluded artery trial (OAT). *Circulation* **2006**, *114*, 2425–2425.
9. Katz, D.L.; Evans, M.A.; Chan, W.; Nawaz, H.; Comerford, B.P.; Hoxey, M.L.; Njike, V.Y.; Sarrel, P.M.
   Oats, antioxidants and endothelial function in overweight, dyslipidemic adults. *J. Am. Coll. Nutr.* **2004**, *23*, 397–403.
10. Rondanelli, M.; Opizzi, A.; Monteferrario, F.; Klersy, C.; Cazzola, R.; Cestaro, B. Beta-glucan- or rice
    bran-enriched foods: A comparative crossover clinical trial on lipidic pattern in mildly hypercholesterolemic men. *Eur. J. Clin. Nutr.* **2011**, *65*, 864–871.
11. Sirtori, C.R.; Triolo, M.; Bosisio, R.; Bondioli, A.; Calabresi, L.; de Vergori, V.; Gomaraschi, M.; Mombelli, G.; Pazzucconi, F.; Zacherl, C.; *et al*. Hypocholesterolaemic effects of lupin protein and pea protein/fibre combinations in moderately hypercholesterolaemic individuals. *Br. J. Nutr.* **2012**, *107*, 1176–1183.
12. Themistoclakis, S.; Corrado, A.; Marchlinski, F.E.; Jais, P.; Zado, E.; Rossillo, A.; di Biase, L.; Schweikert, R.A.; Saliba, W.I.; Horton, R.; *et al*. The Risk of Thromboembolism and Need for Oral Anticoagulation After Successful Atrial Fibrillation Ablation. *J. Am. Coll. Cardiol.* **2010**, *55*, 735–743.
13. Tighe, P.; Duthie, G.; Brittenden, J.; Vaughan, N.; Mutch, W.; Simpson, W.G.; Duthie, S.; Horgan, G.W.; Thies, F. Effects of Wheat and Oat-Based Whole Grain Foods on Serum Lipoprotein Size and Distribution in Overweight Middle Aged People: A Randomised Controlled Trial. *PLoS ONE* **2013**, *8*, e70436,
14. Anderson, J.W.; Hanna, T.J. Impact of nondigestible carbohydrates on serum lipoproteins and risk for cardiovascular disease. *J. Nutr.* **1999,** *129*, 1457S–1466S.
15. Benavente, L.; Calleja, S.; de la Vega, V.; García, J.; Lahoz, C.H. Oral anticoagulation in elderly patients as secondary prevention of cardioembolic strokes. *Int. Arch. Med.* **2010,** *3*, doi:10.1186/1755-7682-3-8.
16. Hlebowicz, J.; Wickenberg, J.; Fahlström, R.; Björgell, O.; Almér, L.O.; Darwiche, G. Effect of commercial breakfast fibre cereals compared with corn flakes on postprandial blood glucose, gastric emptying and satiety in healthy subjects: A randomized blinded crossover trial. *Nutr. J.* **2007**, *6*, **doi:**10.1186/1475-2891-6-22.
17. Jacobs, D.R., Jr.; Pereira, M.A.; Stumpf, K.; Pins, J.J.; Adlercreutz, H. Whole grain food intake elevates serum enterolactone. *Br. J. Nutr.* **2002**, *88*, 111–116.
18. Laaksonen, D.E.; Toppinen, L.K.; Juntunen, K.S.; Autio, K.; Liukkonen, K.H.; Poutanen, K.S.; Niskanen, L.; Mykkänen, H.M. Dietary carbohydrate modification enhances insulin secretion in persons with the metabolic syndrome. *Am. J. Clin. Nutr.* **2005**, *82*, 1218–1227.
19. Lankinen, M.; Schwab, U.; Gopalacharyulu, P.V.; Seppänen-Laakso, T.; Yetukuri, L.; Sysi-Aho, M.; Kallio, P.; Suortti, T.; Laaksonen, D.E.; Gylling, H.; *et al*. Dietary carbohydrate modification alters serum metabolic profiles in individuals with the metabolic syndrome. *Nutr. Metab. Cardiovasc. Dis.* **2010**, *20*, 249–257.
20. Norris, J.M.; Barriga, K.; Hoffenberg, E.J.; Taki, I.; Miao, D.; Haas, J.E.; Emery, L.M.; Sokol, R.J.; Erlich, H.A.; Eisenbarth, G.S.; *et al*. Risk of celiac disease autoimmunity and timing of gluten introduction in the diet of infants at increased risk of disease. *J. Am. Med. Assoc.* **2005**, *293*, 2343–2351.
21. Overgaard, C.B.; Džavík, V.; Buller, C.E.; Liu, L.; Banasiak, W.; Devlin, G.; Maggioni, A.P.; Leor, J.;
    Burton, J.R.; Reis, G.; *et al*. Percutaneous revascularization and long term clinical outcomes of diabetic patients randomized in the Occluded Artery Trial (OAT). *Int. J. Cardiol.* **2013**, *168*, 2416–2422.
22. Panahi, S.; Ezatagha, A.; Temelli, F.; Vasanthan, T.; Vuksan, V. β-glucan from two sources of oat concentrates affect postprandial glycemia in relation to the level of viscosity. *J. Am. Coll. Nutr.* **2007**, *26*, 639–644.
23. Poli, D.; Antonucci, E.; Grifoni, E.; Abbate, R.; Gensini, G.F.; Prisco, D. Stroke risk in atrial fibrillation patients on warfarin: Predictive ability of risk stratification schemes for primary and secondary prevention. *Thromb. Haemost.* **2009**, *101*, 367–372.
24. Roberts, D.C.K.; Truswell, A.S.; Bencke, A.; Dewar, H.M.; Farmakalidis, E. The cholesterol-lowering effect of a breakfast cereal containing psyllium fibre. *Med. J. Aust.* **1994**, *161*, 660–664.
25. Valachovičová, M.; Krajčovičová-Kudláčková, M.; Blažíček, P.; Babinská, K. No evidence of insulin resistance in normal weight vegetarians: A case control study. *Eur. J. Nutr.* **2006**, *45*, 52–54.
26. Weickert, M.O.; Mohlig, M.; Koebnick, C.; Holst, J.J.; Namsolleck, P.; Ristow, M.; Osterhoff, M.; Rochlitz, H.; Rudovich, N.; Spranger, J.; *et al*. Impact of cereal fibre on glucose-regulating factors. *Diabetologia* **2005**, *48*, 2343–2353.
27. Weickert, M.O.; Möhlig, M.; Schöfl, C.; Arafat, A.M.; Otto, B.; Viehoff, H.; Koebnick, C.; Kohl, A.; Spranger, J.; Pfeiffer, A.F.H. Cereal fiber improves whole-body insulin sensitivity in overweight and obese women. *Diabetes Care* **2006**, *29*, 775–780.
28. Weickert, M.O.; Spranger, J.; Holst, J.J.; Otto, B.; Koebnick, C.; Möhlig, M.; Pfeiffer, A.F.H. Wheat-fibre-induced changes of postprandial peptide YY and ghrelin responses are not associated with acute alterations of satiety. *Br. J. Nutr.* **2006**, *96*, 795–798.
29. White, H.D.; Reynolds, H.R.; Carvalho, A.C.; Pearte, C.A.; Liu, L.; Martin, C.E.; Knatterud, G.L.; Džavík, V.; Kruk, M.; Steg, P.G.; *et al*. Reinfarction after percutaneous coronary intervention or medical management using the universal definition in patients with total occlusion after myocardial infarction: Results from long-term follow-up of the Occluded Artery Trial (OAT) cohort. *Am. Heart J.* **2012**, *163*, 563–571.
30. Wolever, T.M.S.; Bolognesi, C. Time of day influences relative glycaemic effect of foods. *Nutr. Res.* **1996**, *16*, 381–384.
31. Wolever, T.M.S.; Vuksan, V.; Palmason, C. Less variation of postprandial blood glucose after starchy test meals than oral glucose. *Nutr. Res.* **1996**, *16*, 899–905.
32. Xiang, W.; Zhang, J.; Liu, M.; Liu, F.; Feng, X.; Wang, Y. Antithrombotic therapy in elderly patients with non-valvular atrial fibrillation: A pilot study. *Clin. Interv. Aging* **2015**, *10*, 515–519.
33. Gu, J.; Jing, L.; Ma, X.; Zhang, Z.; Xu, M.; Wang, J.; Li, Y. Naked oat combined with a structured dietary intervention affects oxidative stress but not inflammation in diabetic dyslipidemia. *Nutr. Metab. Cardiovasc. Dis.* **2014**, *24*, 35–37.
34. Maki, K.C.; Galant, R.; Samuel, P.; Tesser, J.; Witchger, M.S.; Ribaya-Mercado, J.D.; Blumberg, J.B.;
    Geohas, J. Effects of consuming foods containing oat beta-glucan on blood pressure, carbohydrate metabolism and biomarkers of oxidative stress in men and women with elevated blood pressure. *Eur. J. Clin. Nutr.* **2007**, *61*, 786–795.
35. Jenkins, D.J.; Nguyen, T.H.; Kendall, C.W.; Faulkner, D.A.; Bashyam, B.; Kim, I.J.; Ireland, C.; Patel, D.; Vidgen, E.; Josse, A.R.; *et al*. The effect of strawberries in a cholesterol-lowering dietary portfolio. *Metabolism* **2008**, *57*, 1636–1644.
36. Ballesteros, M.; Valenzuela, F.; Robles, A.; Artalejo, E.; Valdez, H.; Fernandez, M.L. One egg a day does not increase the risk for cardiovascular disease in diabetic patients. *FASEB J.* **2014**, *28*, 381–385.
37. Sichert-Oevermann, W.; Koerber, K.; Bretthauer, B.; Leitzmann, C.; Laube, H. Blood glucose and
    insulin levels in healthy persons and diabetics after intake of coarse wholemeal preparations, especially fresh grain muesli. *Deutsch. Med. Wochenschr. (1946)* **1987**, *112*, 1977–1983.
38. Keogh, G.F.; Cooper, G.J.S.; Mulvey, T.B.; McArdle, B.H.; Coles, G.D.; Monro, J.A.; Poppitt, S.D. Randomized controlled crossover study of the effect of a highly β-glucan-enriched barley on cardiovascular disease risk factors in mildly hypercholesterolemic men. *Am. J. Clin. Nutr.* **2003**, *78*, 711–718.
39. Mootoosamy, A.; Fawzi Mahomoodally, M. Ethnomedicinal application of native remedies used against diabetes and related complications in Mauritius. *J. Ethnopharmacol.* **2014**, *151*, 413–444.
40. Poppitt, S.D.; van Drunen, J.D.E.; McGill, A.T.; Mulvey, T.B.; Leahy, F.E. Supplementation of a
    high-carbohydrate breakfast with barley β-glucan improves postprandial glycaemic response for meals
    but not beverages. *Asia Pac. J. Clin. Nutr.* **2007**, *16*, 16–24.
41. Kallio, P.; Kolehmainen, M.; Laaksonen, D.E.; Kekäläinen, J.; Salopuro, T.; Sivenius, K.; Pulkkinen, L.; Mykkänen, H.M.; Niskanen, L.; Uusitupa, M.; *et al*. Dietary carbohydrate modification induces alterations in gene expression in abdominal subcutaneous adipose tissue in persons with the metabolic syndrome:
    The FUNGENUT Study. *Am. J. Clin. Nutr.* **2007**, *85*, 1417–1427.
42. Kallio, P.; Kolehmainen, M.; Laaksonen, D.E.; Pulkkinen, L.; Atalay, M.; Mykkänen, H.; Uusitupa, M.; Poutanen, K.; Niskanen, L. Inflammation markers are modulated by responses to diets differing in postprandial insulin responses in individuals with the metabolic syndrome. *Am. J. Clin. Nutr.* **2008**, *87*, 1497–1503.
43. Zhang, X.; McGeoch, S.C.; Megson, I.L.; MacRury, S.M.; Johnstone, A.M.; Abraham, P.; Pearson, D.W.M.; de Roos, B.; Holtrop, G.; O’Kennedy, N.; *et al*. Oat-enriched diet reduces inflammatory status assessed
    by circulating cell-derived microparticle concentrations in type 2 diabetes. *Mol. Nutr. Food Res.* **2014**, *58*, 1322–1332.
44. Guevara-Cruz, M.; Tovar, A.R.; Aguilar-Salinas, C.; Medina-Vera, I.; Gil-Zenteno, L.; Hernández-Viveros, I.; López-Romero, P.; Ordaz-Nava, G.; Canizales-Quinteros, S.; Pineda, L.E.G.; *et al*. A dietary pattern including nopal, chia seed, soy protein, and oat reduces serum triglycerides and glucose intolerance in patients with metabolic syndrome. *J. Nutr.* **2012**, *142*, 64–69.
45. Hajifaraji, M.; Najjar Safari, S.; Rezvani, V.; Rashidkhani, B.; Maddah, M. Comparison study between the effect of oat and barley breads on serum glucose and lipid profiles in dyslipidemic and type 2 diabetic subjects: A short-term trial. *Mediterr. J. Nutr. Metab.* **2012**, *5*, 247–252.
46. Moustafa, T.A.; Kamel, H.S.; el Malt, M.A. High dietary fibre intake (Talbina) as adjunct in the management of diabetic macular edema. *J. Med. Sci.* **2007**, *7*, 81–87.
47. Nor Munirah, M.Y.; Siti Shafurah, A.; Norazmir, M.N.; Hayati Adilin, M.A.M.; Ajau, D. Roles of whole grains-based products in maintaining treatment targets among Type 2 diabetes mellitus patients. *Asian J. Clin. Nutr.* **2012**, *4*, 67–76.
48. Tan, S.L.; Juliana, S.; Sakinah, H. Dietary compliance and its association with glycemic control among poorly controlled type 2 diabetic outpatients in Hospital Universiti Sains Malaysia. *Malays. J. Nutr.* **2011**, *17*, 287–299.

1. Virtanen, S.M.; Takkinen, H.M.; Nevalainen, J.; Kronberg-Kippilä, C.; Salmenhaara, M.; Uusitalo, L.; Kenward, M.G.; Erkkola, M.; Veijola, R.; Simell, O.; *et al*. Early introduction of root vegetables in infancy associated with advanced ß-cell autoimmunity in young children with human leukocyte antigen-conferred susceptibility to Type 1 diabetes. *Diabet. Med.* **2011**, *28*, 965–971.
2. Zerm, R.; Helbrecht, B.; Jecht, M.; Hein, A.; Millet, E.; Girke, M.; Kröz, M. Oatmeal diet days may improve insulin resistance in patients with type 2 diabetes mellitus. *Forsch. Komplementarmed.* **2013**, *20*, 465–468.
3. Greenway, F.; Wang, S.; Heiman, M. A novel cobiotic containing a prebiotic and an antioxidant augments the glucose control and gastrointestinal tolerability of metformin: A case report. *Benef. Microbes* **2014**, *5*,
   29–32.
4. Reyna, N.Y.; Cano, C.; Bermúdez, V.J.; Medina, M.T.; Souki, A.J.; Ambard, M.; Nuñez, M.; Ferrer, M.A.; Inglett, G.E. Sweeteners and beta-glucans improve metabolic and anthropometrics variables in well controlled type 2 diabetic patients. *Am. J. Ther.* **2003**, *10*, 438–443.
5. Yu, K.; Ke, M.Y.; Li, W.H.; Zhang, S.Q.; Fang, X.C. The impact of soluble dietary fibre on gastric emptying, postprandial blood glucose and insulin in patients with type 2 diabetes. *Asia Pac. J. Clin. Nutr.* **2014**, *23*, 210–218.
6. Tapola, N.; Karvonen, H.; Niskanen, L.; Mikola, M.; Sarkkinen, E. Glycemic responses of oat bran products in type 2 diabetic patients. *Nutr. Metab. Cardiovasc. Dis.* **2005**, *15*, 255–261.
7. Jenkins, A.L.; Jenkins, D.J.A.; Zdravkovic, U.; Würsch, P.; Vuksan, V. Depression of the glycemic index by high levels of β-glucan fiber in two functional foods tested in type 2 diabetes. *Eur. J. Clin. Nutr.* **2002**, *56*, 622–628.
8. McGeoch, S.C.; Johnstone, A.M.; Lobley, G.E.; Adamson, J.; Hickson, K.; Holtrop, G.; Fyfe, C.; Clark, L.F.; Pearson, D.W.M.; Abraham, P.; *et al*. A randomized crossover study to assess the effect of an oat-rich diet on glycaemic control, plasma lipids and postprandial glycaemia, inflammation and oxidative stress in
   Type 2 diabetes. *Diabet. Med.* **2013**, *30*, 1314–1323.
9. Pick, M.E.; Hawrysh, Z.J.; Gee, M.I.; Toth, E.; Garg, M.L.; Hardin, R.T. Oat bran concentrate bread products improve long-term control of diabetes: A pilot study. *J. Am. Diet. Assoc.* **1996**, *96*, 1254–1261.
10. Asmaa, B.; Riffat, P.; Samia, K. Nutritional composition and metabolic effects of oat dietary fiber extracts on diabetic and hypercholesterolemic male rats. *Pak. J. Nutr.* **2014**, *13*, 527–532.
11. Cervantes-Pahm, S.K.; Liu, Y.; Stein, H.H. Comparative digestibility of energy and nutrients and fermentability of dietary fiber in eight cereal grains fed to pigs. *J. Sci. Food Agric.* **2014**, *94*, 841–849.
12. Xu, C.; Zhu, L.; Chan, T.; Lu, X.; Shen, W.; Gillies, M.C.; Zhou, F. The altered renal and hepatic expression of Solute carrier transporters (SLCs) in type 1 diabetic mice. *PLoS ONE* **2015**, *10*, e0120760.
13. Youn, M.; Saari Csallany, A.; Gallaher, D.D. Whole grain consumption has a modest effect on the development of diabetes in the Goto-Kakisaki rat. *Br. J. Nutr.* **2012**, *107*, 192–201.
14. Bao, L.; Cai, X.; Xu, M.; Li, Y. Effect of oat intake on glycaemic control and insulin sensitivity: A meta-analysis of randomised controlled trials. *Br. J. Nutr.* **2014**, *112*, 457–466.
15. Ha, V.; Sievenpiper, J.L.; de Souza, R.J.; Jayalath, V.H.; Mirrahimi, A.; Agarwal, A.; Chiavaroli, L.;
    Mejia, S.B.; Sacks, F.M.; di Buono, M.; *et al*. Effect of dietary pulse intake on established therapeutic lipid targets for cardiovascular risk reduction: A systematic review and meta-analysis of randomized controlled trials. *Can. Med. Assoc. J.* **2014**, *186*, E252–E262.
16. Ballesteros, M.N.; Valenzuela, F.; Robles, A.E.; Artalejo, E.; Aguilar, D.; Andersen, C.J.; Valdez, H.; Fernandez, M.L. One egg per day improves inflammation when compared to an oatmeal-based breakfast without increasing other cardiometabolic risk factors in diabetic patients. *Nutrients* **2015**, *7*, 3449–3463.
17. Cavazos, A.; Gonzalez de Mejia, E. Identification of Bioactive Peptides from Cereal Storage Proteins and Their Potential Role in Prevention of Chronic Diseases. *Compr. Rev. Food Sci. Food Saf.* **2013**, *12*, 364–380.
18. Cloetens, L.; Ulmius, M.; Johansson-Persson, A.; Åkesson, B.; Önning, G. Role of dietary beta-glucans in the prevention of the metabolic syndrome. *Nutr. Rev.* **2012**, *70*, 444–458.
19. Jonnalagadda, S.S.; Harnack, L.; Liu, R.H.; McKeown, N.; Seal, C.; Liu, S.; Fahey, G.C. Putting the whole Grain Puzzle Together: Health benefits associated with whole grains-summary of American Society for Nutrition 2010 satellite symposium. *J. Nutr.* **2011,** *141*, 1011S–1022S.
20. Khan, M.I.; Anjum, F.M.; Sohaib, M.; Sameen, A. Tackling metabolic syndrome by functional foods.
    *Rev. Endocr. Metab. Disord.* **2013**, *14*, 287–297.
21. Othman, R.A.; Moghadasian, M.H.; Jones, P.J.H. Cholesterol-lowering effects of oat β-glucan. *Nutr. Rev.* **2011**, *69*, 299–309.
22. Pandian, R.S. Functional foods in managing diabetes. *Int. J. Pharma Bio Sci.* **2013**, *4*, B572–B579.
23. Strazzullo, P.; Giacco, R. Fibre intake and blood pressure: More facts, more questions. *J. Hypertens.* **2015**, *33*, 921–923.
24. Tiwari, U.; Cummins, E. Meta-analysis of the effect of β-glucan intake on blood cholesterol and glucose levels. *Nutrition* **2011**, *27*, 1008–1016.
25. Whitehead, A.; Beck, E.J.; Tosh, S.; Wolever, T.M.S. Cholesterol-lowering effects of oat β-glucan: A meta-analysis of randomized controlled trials1. *Am. J. Clin. Nutr.* **2014**, *100*, 1413–1421.
26. Wolever, T.M.S. Do whole grain cereals really reduce LDL cholesterol by 0.72 mmol/L? *J. Nutr.* **2013**, *143*, 1521–1522.
27. Rezvani, V.; Najjar Safari, S.; Hajifaraji, M.; Rashidkhani, B. The effect of “real oat bread” compared with “barley bread offered in Tehran” on serum glucose and lipid profiles in dislipidemic and type 2 diabetic subjects. *Iran. J. Endocrinol. Metab.* **2011**, *13*, 233–242.
